# Treatment of an Infected Tibial Shaft Non-Union Using a Novel 3D-Printed Titanium Mesh Cage: A Case Report

**DOI:** 10.7759/cureus.34212

**Published:** 2023-01-25

**Authors:** Kevin Tang, Wesley Day, Sandip Tarpada, Mani D Kahn

**Affiliations:** 1 Department of Orthopaedic Surgery, Albert Einstein College of Medicine, The Bronx, USA; 2 Department of Orthopaedic Surgery, Montefiore Medical Center, The Bronx, USA

**Keywords:** non-union, endoprosthesic reconstruction, critical sized defect, 3d printing, tibial shaft non-union

## Abstract

Treating large bone defects resulting from trauma, tumors, or infection can be challenging, as current methods such as external fixation with bone transport, bone grafting, or amputation often come with high costs, high failure rates, high requirements for follow-up, and potential complications. In this case report, we present the successful treatment of a complicated, infected tibial shaft non-union by using a personalized three-dimensional (3D)-printed titanium mesh cage. This case adds to the existing body of literature by demonstrating successful integration of bone into a titanium implant and a demonstration of immediate postoperative weight bearing in the setting of suboptimal operative and psychosocial conditions. Futhermore, this report highlights the flexibility of 3D-printing technology and its ability to allow for continued limb salvage, even after a planned bone transport procedure has been interrupted. The use of 3D-printed implants customized to the patient's specific needs offers a promising new avenue for treating complex tibial pathologies, and the technology's versatility and ability to be tailored to individual patients makes it a promising tool for addressing a wide range of bone defects.

## Introduction

Three-dimensional (3D) printing has widespread applications in orthopaedic surgery. While several reports have demonstrated the utility of 3D printing, this technology remains in the early stages of adoption [1,2]. One potential application is the treatment of bone defects caused by trauma, tumors, or infection. There is a lack of consensus regarding the optimal management of bone defects, and several popular methods are discussed here.

Direct bone grafting is typically limited to smaller (<5cm) or partial bone defects but has limitations related to donor site morbidity, infection, non-union, and refracture [3-7]. The Masquelet technique is a two-stage procedure thought to enhance the effectiveness of grafting with the formation of a privileged biologically active compartment starting with debridement and implantation of a temporary cement spacer, followed by spacer removal and bone grafting after the formation of a “pseudomembrane" [8]. The Ilizarov method of distraction osteogenesis utilizes the bone’s natural capacity to regenerate under tension, and is employed in bone transport techniques [9]. The benefits include maintenance of limb length and function and effective treatment of large defects, without donor site morbidity [10]. However, this method is notoriously challenging for patients and surgeons alike and is associated with a high rate of complications [9].

Endoprosthetic reconstruction is feasible when the bone defect traverses an adjacent joint. An increasingly popular and evolving treatment method includes the use of custom 3D-printed implants. These can be aimed at producing segmental bone growth or bone ingrowth at the ends of the prosthetic. The use of this method in critical bone loss has been reported with promising results [11-13].

Here, we present a patient with a significant segmental bony defect of the tibial shaft treated with the novel use of a 3D-printed titanium mesh cage integrated with an intramedullary nail.

## Case presentation

**Figure 1:**
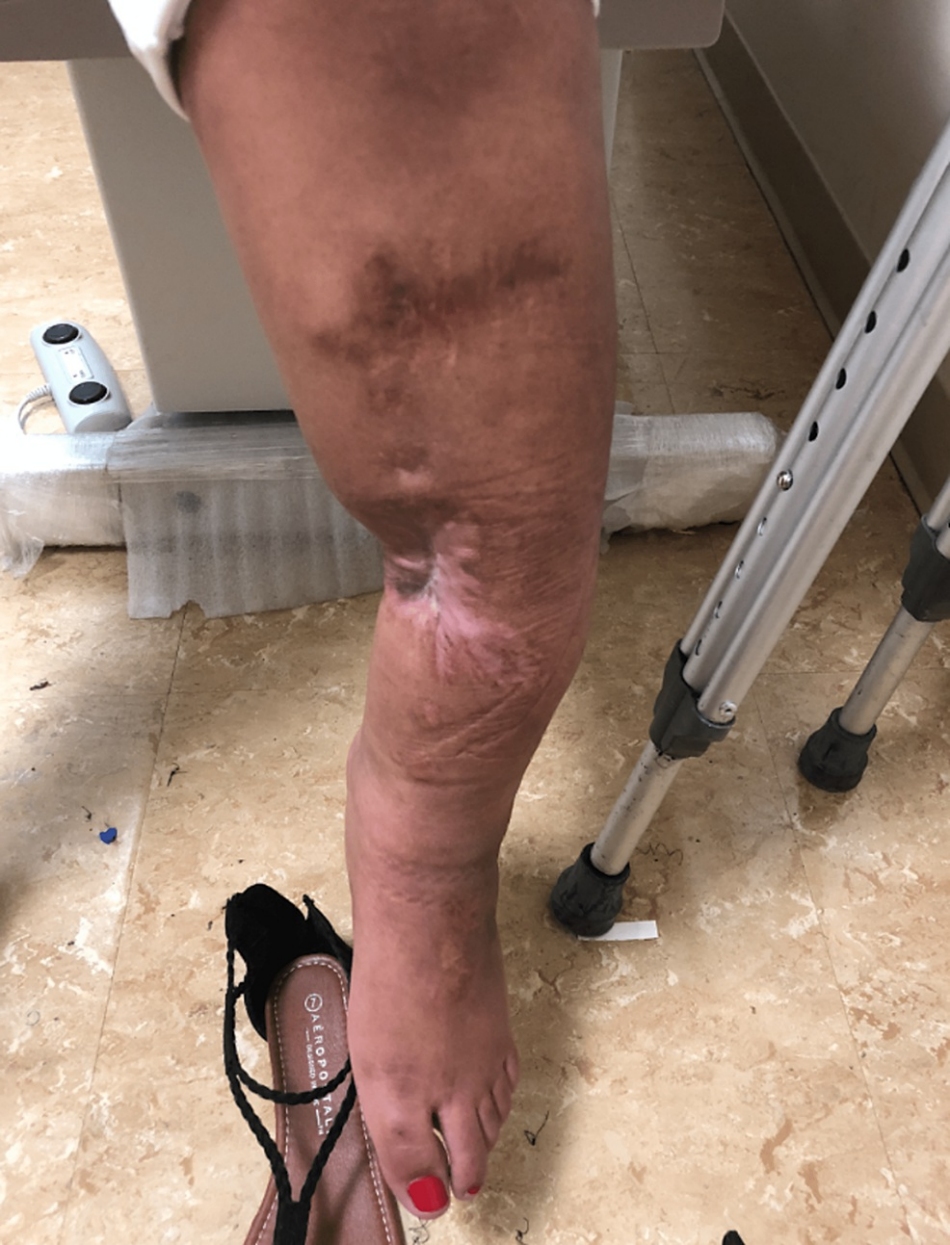
Evaluation visit photograph showing a left tibia with varus malalignment.

**Figure 2:**
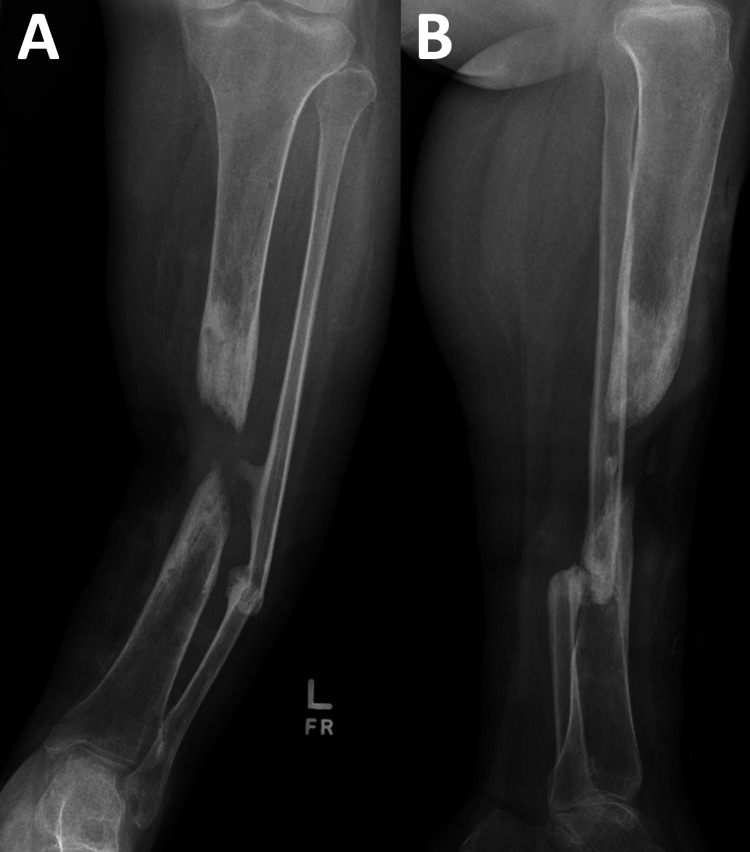
(A) Anteroposterior and (B) lateral radiographs of a left tibia non-union demonstrating a significant bony defect with varus malalignment and diffuse osteopenia.

A 25-year-old female presented to our orthopaedic trauma service for evaluation of an infected left tibial shaft non-union with a 5 cm bone defect, 6 cm limb length discrepancy, and varus malalignment (Figures 1, 2). She sustained an open left tibial shaft fracture from a motor vehicle accident treated overseas with the placement of a definitive uniplanar external fixator two years prior. After external fixator removal, she developed an infected non-union (*Klebsiella oxytoca*) and was treated with isolated debridement and antibiotics.

**Figure 3:**
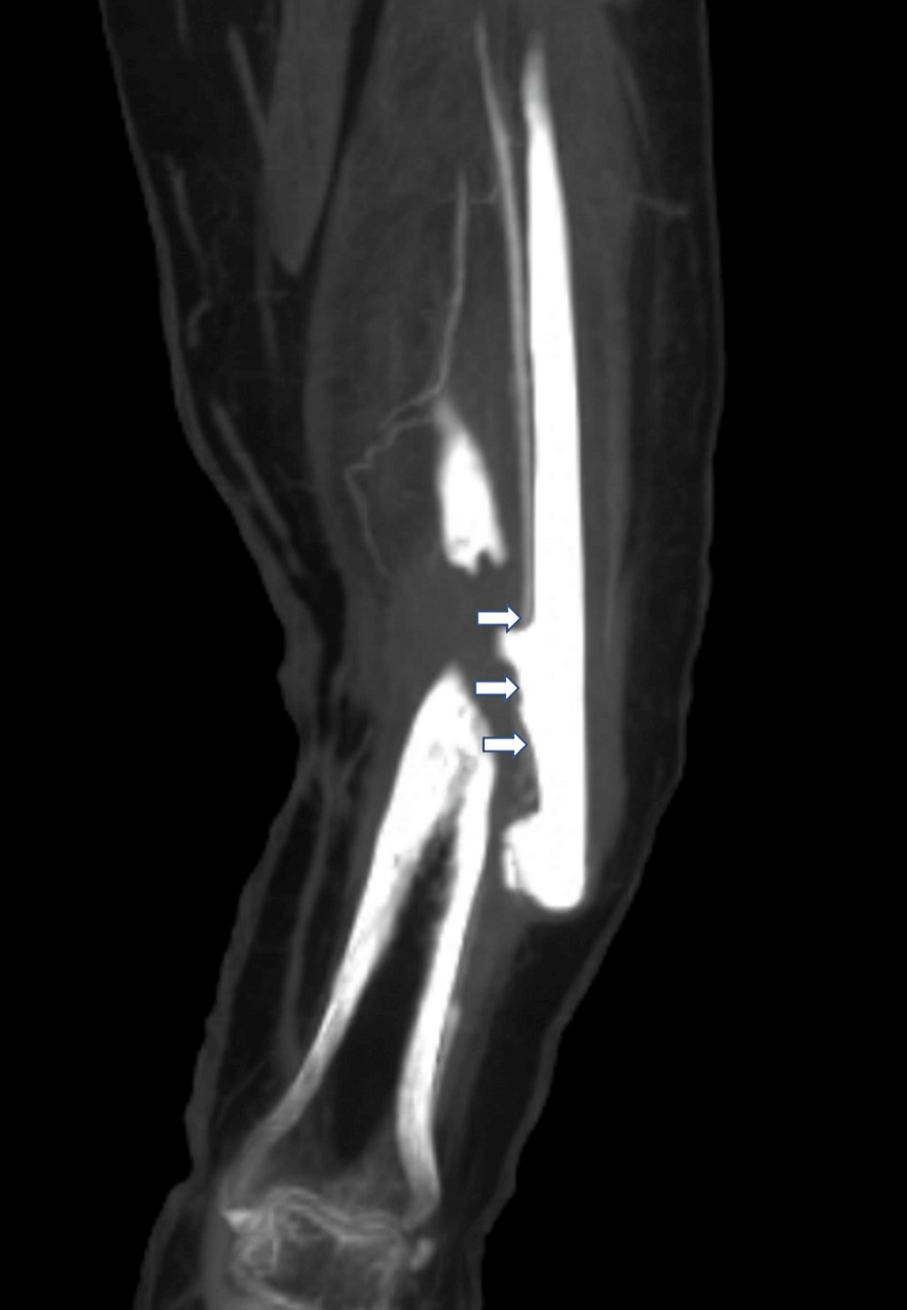
CT angiography revealing occlusion of left anterior tibial artery near the level of defect.

**Figure 4:**
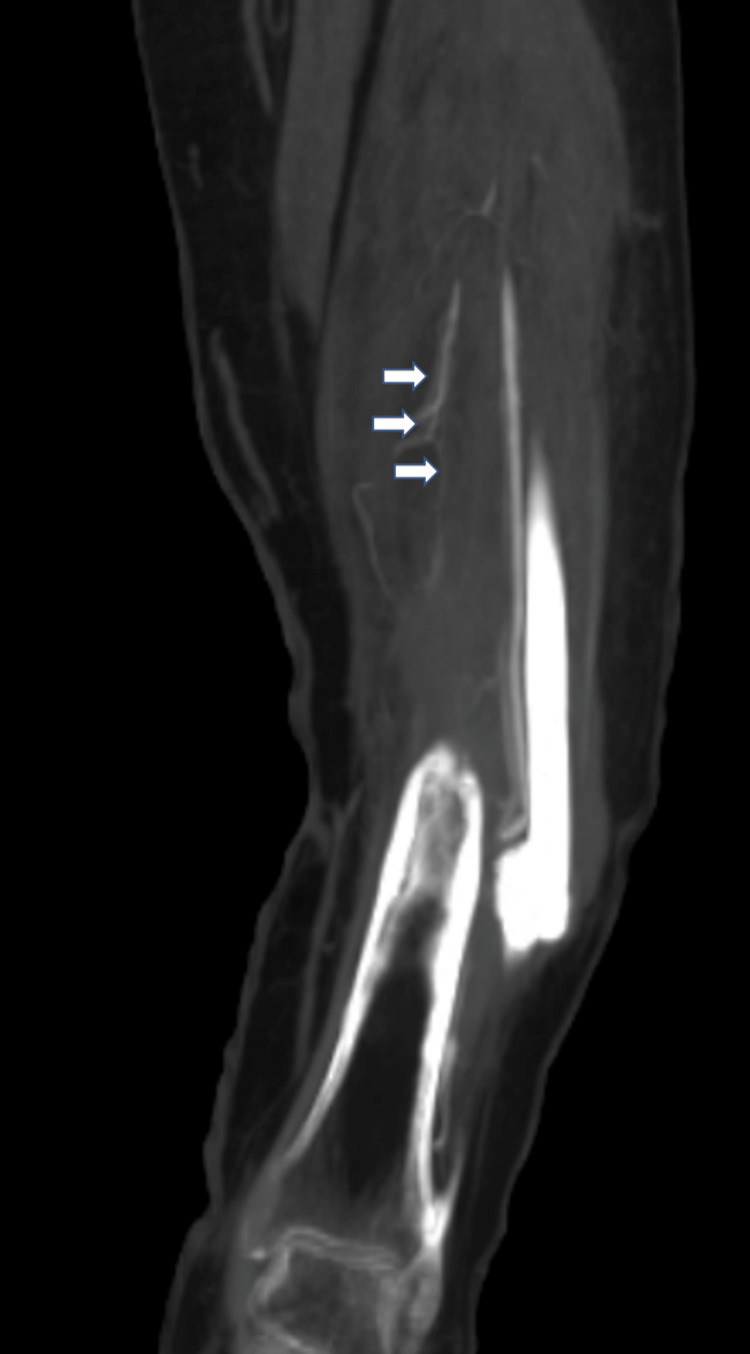
CT angiography revealing occlusion of left posterior tibial artery near the level of defect.

An initial examination of her left leg revealed contracted soft tissues invaginated into the bone defect, a flexible varus deformity, intact distal neurological function, and a warm foot without pulses. The patient was crutch-dependent since her initial injury. CT angiography demonstrated complete occlusion of both her anterior and posterior tibial arteries (Figures 3, 4) at the level of the fracture and MRI revealed persistent tibial osteomyelitis. 

**Figure 5:**
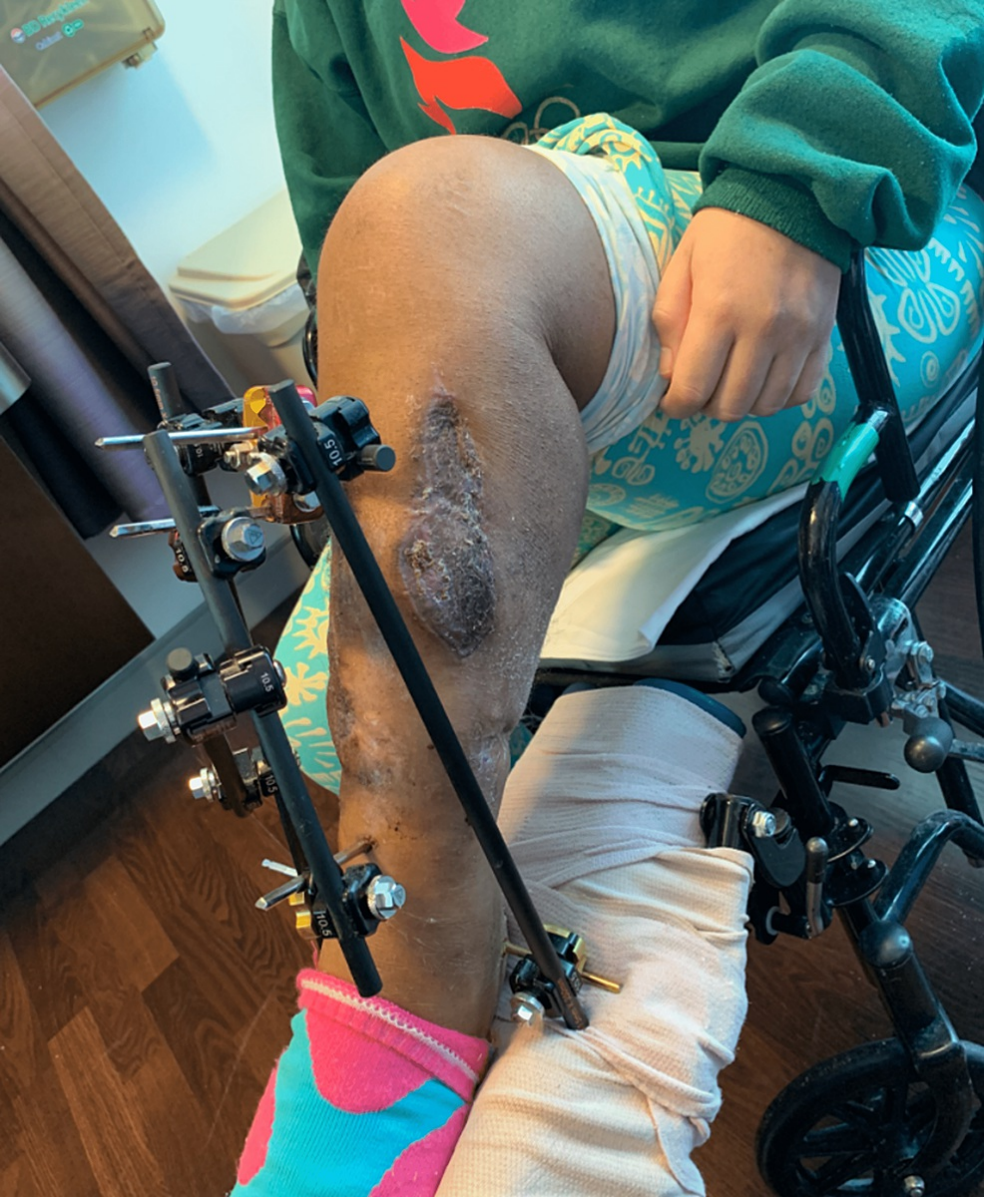
Postoperative photographs of the patient’s leg after placement of a cement spacer and external fixator.

**Figure 6:**
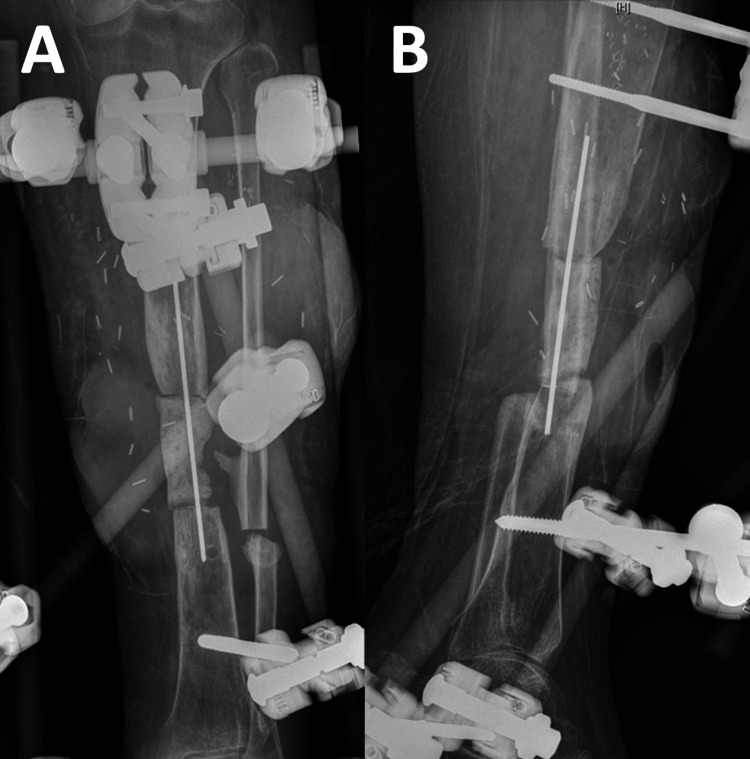
Postoperative (A) anteroposterior and (B) lateral radiographs of the left tibia after placement of a cement spacer and external fixator.

Of note, the patient was undomiciled and did not have any family or peer support. Amputation was discussed as the most rapid method of restoring function; however, she requested all efforts be directed at limb salvage. Given the complexity of limb salvage with staged free flap and bone transport using a ring fixator, social work was engaged and surgery was deferred until the patient had her living situation addressed. She then underwent irrigation and debridement of the left tibia, placement of an antibiotic cement spacer, rectus abdominus free flap, and static external fixation (Figures 5, 6), with a plan to return to initiate bone transport after flap maturation.

After the operation, the flap remained viable, cultures were found to be negative and she was treated with 11 days of intravenous cefazolin. Unfortunately, during her hospitalization, the patient developed a sudden onset of altered mental status including severe agitation, hallucinations, disorganized thoughts, and tangential speech. She was later diagnosed with paraneoplastic encephalitis due to an ovarian teratoma. Due to the patient’s psychosocial impediments, poor reliability, lack of family and peer support, and the ongoing coronavirus disease 2019 (COVID-19) pandemic, the decision was made to abort the plan for bone transport for limb salvage. Amputation was once again weighed, and the patient requested the simplest limb salvage solution. 

With the infection treated, the decision was made to proceed with definitive fixation with structural void-filling hardware that would allow immediate weight-bearing postoperatively and alleviate the need for patient or family involvement in the execution of care. This also left open the possibility for bone transport in the future should the patient's psychosocial condition improve. After careful deliberation, the proposed construct for surgery included a statically locked intramedullary nail, cannulated 3D-printed titanium mesh cage, and bone grafting.

In collaboration with Restor3D Inc. (Durham, North Carolina, United States), CT imaging of the patient's leg within the external fixator was used to render 3D images for preoperative planning as well as manufacturing a customized 3D-printed titanium mesh cage. The patient’s preoperative anatomy, proposed bone resection, and implant specifications were carefully templated in preparation for her surgery. On the day of the surgery, three titanium alloy implants of varying sizes were available. 

**Figure 7:**
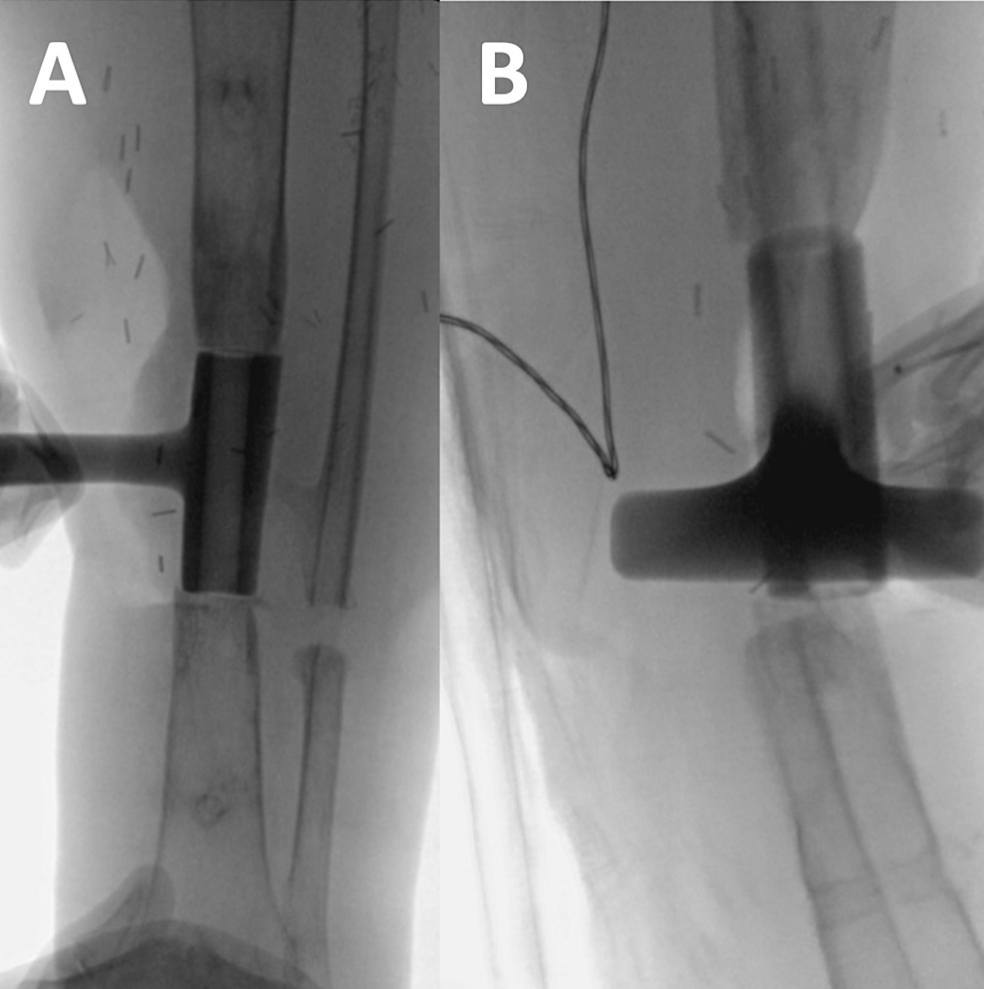
Intra-operative fluoroscopic images demonstrating (A) anteroposterior and (B) lateral positioning of trial implants confirming adequate bony contact and alignment.

**Figure 8:**
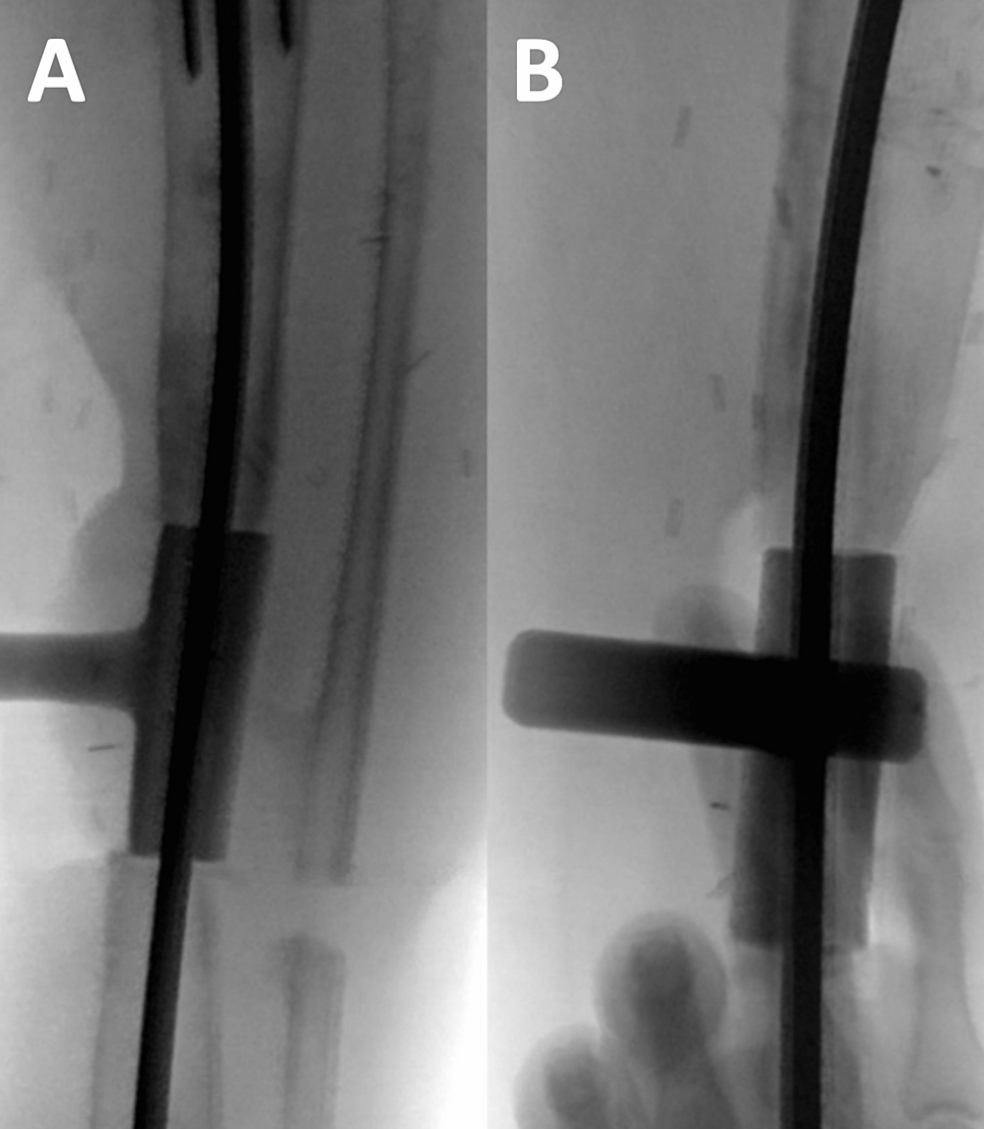
(A) Anteroposterior and (B) lateral views showing reaming of the tibial shaft through the trial implant.

**Figure 9:**
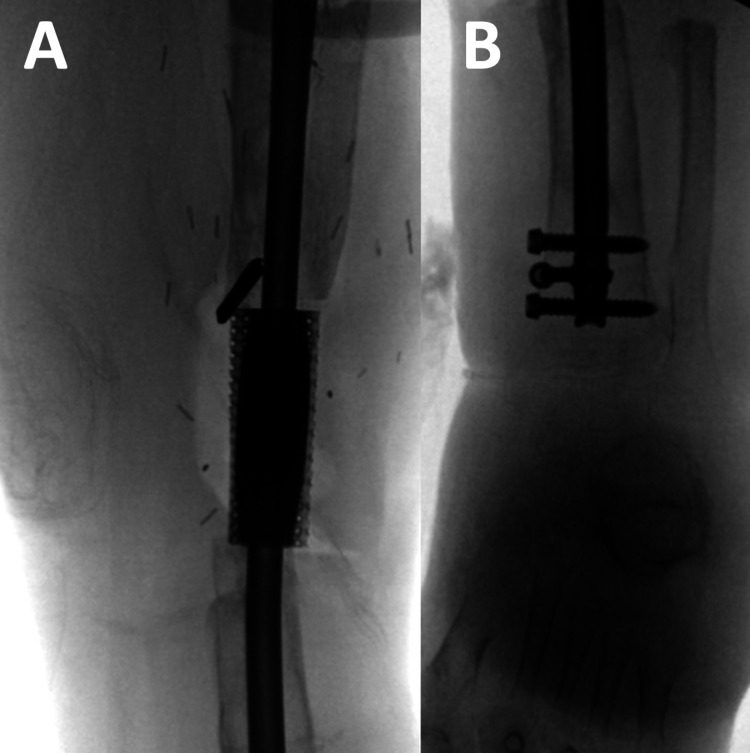
(A) Proximal segment posterior blocking screw and (B) distal segment medial blocking screw ensuring optimal placement of intramedullary tibial nail.

Surgery commenced with elevation of the free flap and cement spacer removal. Bone resection was performed. Next, trials were used to select the largest implant, and the tibial shaft was reamed through the trial. The implant was then placed into the bone defect with interference fit. Using the blocking screw technique both proximal and distal to the segmental defect, an intramedullary nail was inserted and passed through the 3D-printed titanium mesh cage and statically locked (Figures 7, 8, 9). To enhance implant ingrowth, an allograft was placed at the proximal and distal segments of the osseous-implant interface. The free flap was then revised by the plastic surgery team. 

**Figure 10:**
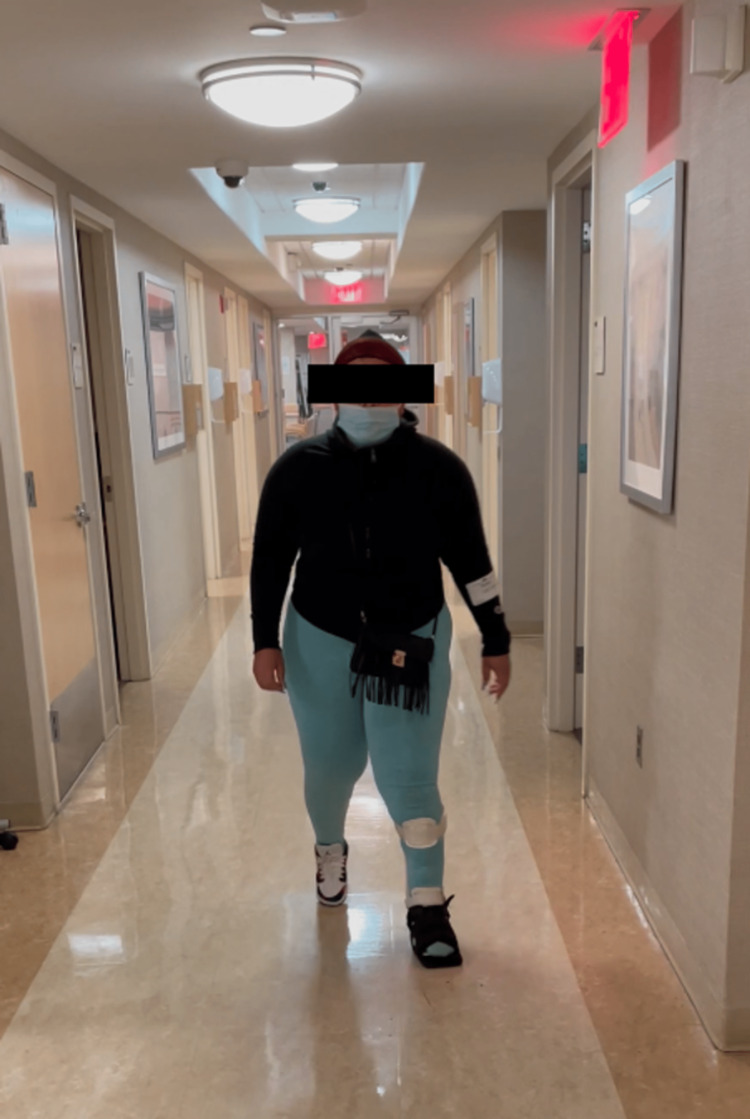
The patient was able to ambulate independently at the one-year follow-up.

**Figure 11:**
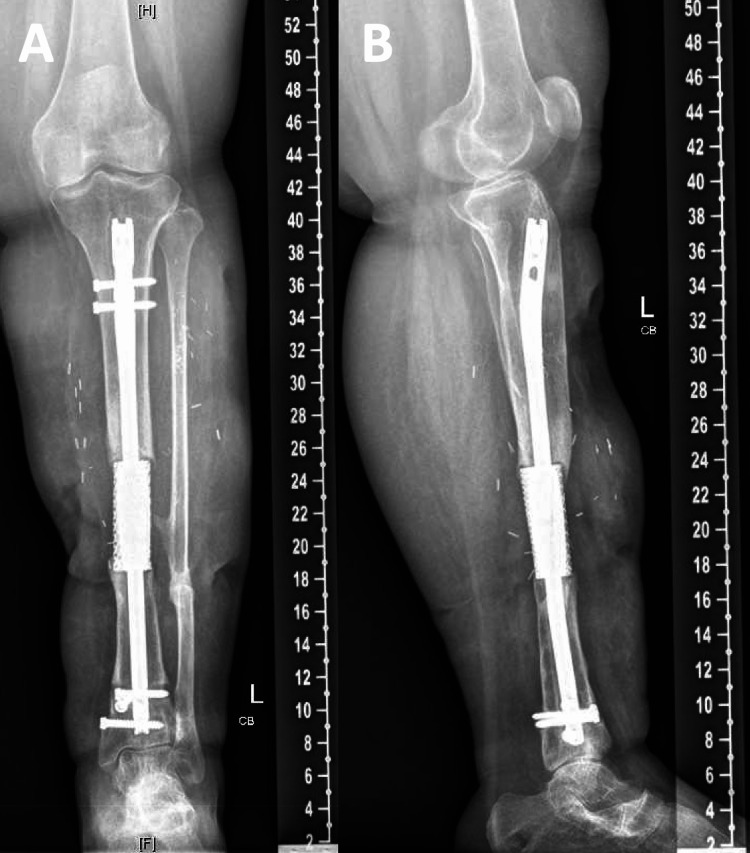
One-year postoperative (A) anteroposterior and (B) lateral radiographs of the left tibia demonstrating 3D-printed titanium implant and intramedullary nail with restored alignment and segmental bony defect. 3D: three-dimensional

The patient’s postoperative course was uncomplicated. She was discharged to a facility in a controlled ankle motion (CAM) boot for stabilization and to facilitate healing. At her one-month postoperative visit, the patient was noted to be independently ambulating for the first time since her injury. Her mental status gradually improved. At the one-year follow-up, the patient reported no pain and remained independent in ambulation using a custom ankle foot orthosis with lift for stabilization (Figure 10). Physical exam revealed healed surgical incisions with no evidence of infection and improved strength and sensation in the extremity. X-rays demonstrated intact hardware at both ends of the 3D implant (Figure 11). 

## Discussion

Three-dimensional printing has been successfully implemented in tibial defects involving the ankle joint [1,11,14]. Nwanko et al. presented a five-year follow-up case of a bone loss of the distal tibia limb salvaged with arthrodesis of the tibia to the hindfoot using a 3D-printed titanium cage and intramedullary rod. CT scan at five years after surgery showed successful bone incorporation of the talus, calcaneus, and tibia with the cage [14]. Similarly, Hsu et al. demonstrated a critical-sized tibial defect with persistent non-union despite external fixation. Successful limb salvage was performed using a 3D-printed titanium truss cage and retrograde intramedullary nail [11].

Our report highlights the potential of 3D printing as a tool in limb salvage, with particular potency to help overcome psychosocial limitations to traditional limb salvage methods. It has been well documented that psychosocial variables can negatively impact postoperative outcomes [15-17]; yet these variables are often underappreciated. A survey of orthopaedic trauma surgeons across 12 centers found that only 50% of providers had “time to ask about psychosocial complications in practice” and only 31.6% of providers had “access to information to guide the management of psychosocial issues related to recovery" [18]. While systemic solutions are essential to equitable care delivery for disadvantaged patients, identification of at-risk patients and coordination of psychosocial support resources, coupled with embracing modern technology, may help reduce complications and optimize outcomes.

The current case report highlights several benefits of utilizing 3D printing technology in the treatment of bone defects. First, it permitted continued limb salvage after interruption of planned bone transport procedure. Second, the custom design and size options of the implant permitted minimal further bone resection to obtain excellent fit and integration with intramedullary fixation. Third, this load-sharing construct allowed for initiation of weight-bearing immediately postoperatively, which served as an important component of her physical and mental recovery. Although this technology is still in the early stages of development, we believe that it has significant potential to facilitate the treatment of complex orthopaedic injuries in the setting of challenging patient psychosocial factors.

## Conclusions

Treating large lower extremity bony defects can be challenging, as these pathologies often require a structural bone void filler of a specific size and shape that may not be available through traditional means such as autograft or allograft bone. One promising solution to this issue is the use of patient-specific 3D-printed titanium implants, which offer flexibility and customization to meet the specific needs of individual patients. The case described here serves as an effective alternative treatment method for tibial shaft non-union in the setting of austere psychosocial conditions. While the surgical techniques employed in this case show promise, it is important to note that they are not yet mainstream and should be used with caution when considering treatment options for other patients.
